# Neuropathological Characterisation of McLeod Syndrome With a Proposed New Grading System

**DOI:** 10.1111/nan.70039

**Published:** 2025-09-02

**Authors:** Anna Maria Reuss, Klavs Renerts, Tibor Hortobágyi, Felix Geser, Johannes Haybaeck, Adrian Danek, Peter Fuhr, Bjarne Udd, Adam Zeman, Reichard R. Ross, Elisabeth J. Rushing, Hans H. Jung

**Affiliations:** ^1^ Institute of Neuropathology University Hospital Zurich, University of Zurich Zurich Switzerland; ^2^ Department of Neurology University Hospital Zurich, University of Zurich Zurich Switzerland; ^3^ Department of Neurology Ludwig‐Maximilian‐University (LMU), University Hospital Munich Munich Germany; ^4^ Depts. of Neurology and of Clinical Research University Hospital Basel Basel Switzerland; ^5^ Neuromuscular Center Tampere University Hospital Tampere Finland; ^6^ Department of Neurology Vaasa Central Hospital Vaasa Finland; ^7^ Department of Clinical Neurosciences University of Edinburgh Edinburgh UK; ^8^ University of Exeter Medical School Exeter UK; ^9^ Department of Laboratory Medicine and Pathology Mayo Clinic Rochester Minnesota USA

**Keywords:** gliosis, histology, movement disorder, neuronal loss, rare disease, vacuoles

## Abstract

**Aims:**

X‐linked McLeod neuroacanthocytosis syndrome (MLS) is a rare neurodegenerative disorder characterised by the presence of red blood cell acanthocytosis and a chorea syndrome. Analogous to Huntington's disease (HD), MLS displays cognitive and behavioural symptoms besides the progressive movement disorder. This study aimed to describe the neuropathology of MLS in the largest case series to date.

**Methods:**

Clinical data were collected, and neuropathological assessments were performed on eight male MLS patients originating from Finland, New Zealand, Switzerland, Scotland and the United States.

**Results:**

Macroscopic data were available from six patients, with five showing atrophy of the basal ganglia, which was more pronounced in the caudate nucleus and to a lesser extent in the putamen and pallidum. Histology revealed neuronal loss and accompanying gliosis in the basal ganglia of all patients. The extent of these alterations varied widely, with a decreasing gradient of severity from the caudate nucleus to the putamen and the pallidum, mirroring the macroscopic findings. In addition, we detected intraneuronal vacuoles in the striatum in half of the patients.

**Conclusions:**

MLS neuropathology is characterised macroscopically by atrophy and microscopically by neuronal loss and gliosis of the basal ganglia, with a decreasing gradient of severity from the caudate nucleus, the putamen to the pallidum. Analogous to the grading system for HD, we propose a neuropathological grading system for MLS based on the current observations in the largest MLS cohort examined to date. Standardised criteria are crucial for neuropathological assessment of this extremely rare disease.

## Summary

- The neuropathology of McLeod syndrome is characterised by neuronal loss, gliosis and atrophy of the basal ganglia.
- There is a severity gradient from the caudate nucleus to the putamen and pallidum, in decreasing order.
- Intraneuronal vacuoles in the basal ganglia of McLeod syndrome patients are a microstructural hallmark of advanced disease.
- Comprehensive investigation of the largest McLeod syndrome patient cohort to date allows for the proposal of a standardised grading system for the neuropathological assessment of McLeod syndrome.

## Graphical abstract

We report the neuropathological findings of the X‐linked McLeod neuroacanthocytosis syndrome (MLS), a very rare neurodegenerative disorder characterised by red blood cell acanthocytosis, chorea syndrome similar to Huntington’s disease, and additional neuromuscular and cardiological involvement. There was varying atrophy of basal ganglia, pronounced in the caudate nucleus. Histologically, we found neuronal loss and accompanying gliosis with additional striatal intraneuronal vacuoles in four of eight patients. Analogous to the HD grading system, we propose a neuropathological grading system for MLS.

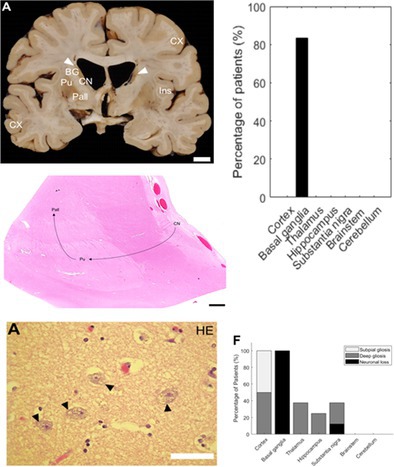

**Abbreviations**
Aβ: amyloid beta
α‐syn: alpha‐synuclein
bp: base pairs
CCT: cerebral computed tomography
CD68: cluster of differentiation 68
ChAc: chorea‐acanthocytosis
CK: creatine phosphokinase
CNS: central nervous system
CV: cresyl violet
DAB: 3,3′‐diaminobenzidine
ECG: electrocardiography
EEG: electroencephalography
EMG: electromyography
ENG: electroneurography
FDG‐PET: (18F)‐2‐fluoro‐2‐deoxy‐glucose‐positron emission tomography
Gall: Gallyas silver
GFAP: glial fibrillary acidic protein
HD: Huntington's disease
HE: haematoxylin and eosin
LFB: Luxol fast blue
MAP 2: microtubule‐associated protein 2
MLS: McLeod syndrome
MRI: magnetic resonance imaging
NF: neurofilament
NFTs: neurofibrillary tangles
p62: protein 62
PART: primary age‐related tauopathy
RBC: red blood cell
SPECT: single photon emission computed tomography
TDP‐43: transactive response DNA binding protein‐43
Ub: ubiquitin

## 1 Introduction

Historically, neuroacanthocytosis syndromes were conceptualised as a collection of rare disorders delineated by a constellation of neurological and/or psychiatric symptoms accompanied by haematological abnormalities, in particular, erythrocyte acanthocytosis (for background and nomenclature, see [1, 2]). McLeod syndrome (MLS or XK disease) and VPS13A disease (formerly chorea‐acanthocytosis; ChAc) are the core syndromes of this group. X‐linked MLS is an extremely rare multisystem disorder with only about 250 cases reported worldwide [3]. The cardinal features include haematological, cardiac, neuromuscular and central nervous system (CNS) involvement. Affected individuals typically show elevated creatine phosphokinase (CK) levels, red blood cell (RBC) acanthocytosis, and absent or diminished tendon reflexes [4, 5, 6]. The CNS manifestations of MLS strongly resemble Huntington's disease (HD), i.e., the triad of movement disorder, psychiatric abnormalities and cognitive alterations, often accompanied by epileptic seizures, and it has a mean onset age of 40 years [4, 5, 6]. Neuromuscular manifestations of MLS include muscle weakness, atrophy and rarely sensory disturbances [4, 7]. The clinical diagnosis can be challenging due to the variable clinical phenotype and similarity to other hyperkinetic diseases, thus requiring additional serologic and/or genetic testing for confirmation. Clinical manifestations of MLS progress slowly but relentlessly, with a mean disease duration of about 30 years [4, 5]. Cardiac complications, including progressive heart failure and sudden death, account for about half of the deaths [4, 7, 8].

The diagnosis of MLS is based on absent expression of the Kx RBC antigen and diminished expression of Kell glycoprotein RBC antigens. Genetic confirmation relies on the detection of a disease‐causing variant in the *XK* gene [3, 9, 10].

Microscopic examination of skeletal muscle demonstrates predominant neurogenic muscle atrophy, axonal sensory–motor neuropathy and variable nonspecific myopathic changes [7]. However, neuropathological data on CNS involvement in MLS patients are sparse and limited to single case reports [11, 12, 13, 14, 15, 16]. Therefore, a neuropathological grading system for a standardised assessment of neuropathological features, as commonly performed for other disease entities, is not yet available. Here, we describe the neuropathological findings in the brains of eight male patients from seven different families with MLS, including the eponymous index patient Hugh McLeod [9]. Investigation of the largest MLS cohort to date has enabled the recognition of consistent morphological patterns that form the basis for a reproducible neuropathological grading system in this extremely rare disease.

## 2 Materials and Methods

### 2.1 Aim, Design and Setting of the Study

The aim of this study was to describe the neuropathology of MLS in the largest case series so far. Clinical data and autopsy material from eight male MLS patients were collected from around the world over a period of 8 years. To account for diagnostic variability, neuropathological assessment of the macroscopy and microscopy of every case was performed by three neuropathologists at the University Hospital Zurich, Switzerland, and the findings were discussed. Correlation with the clinical data was performed in an interdisciplinary manner with two neurologists from the University Hospital Zurich, Switzerland.

### 2.2 Patients

The eight male patients originated from seven families across the globe, including Switzerland, Finland, New Zealand, Scotland and the United States. This study was approved by the local ethics committee in Zurich (BASEC‐Nr. 2022‐006). Seven of the eight cases have been partly described in previous publications [5, 7, 17, 18, 19].

### 2.3 Clinical Examination

Immunohaematological analysis of Kx and Kell expression, as well as peripheral blood smears for acanthocytosis detection and serum CK levels, was performed in all patients. We reviewed all available reports of the clinical examination and neuropsychological testing. We also reviewed data from additional examinations such as echocardiography, electrocardiography (ECG), electroencephalography (EEG), electromyography (EMG), electroneurography (ENG), cerebral computed tomography (CCT), magnetic resonance imaging (MRI), as well as cerebral metabolic studies, i.e., positron emission tomography with (18F)‐2‐fluoro‐2‐deoxy‐glucose (FDG‐PET) and Tc99m exametazime single photon emission computed tomography (SPECT).

### 2.4 Macroscopic Brain Examination

Macroscopic descriptions of formalin‐fixed brains were available in six of the eight cases. Blocks from various brain regions had been sampled, dehydrated and embedded into paraffin according to standard protocols used in routine diagnostics.

### 2.5 Microscopic Brain Examination

Haematoxylin and eosin (HE) stains on brain tissue sections from different brain regions were available for all patients. For some cases, additional histochemical stains such as Gallyas silver (Gall), Luxol fast blue (LFB) and cresyl violet (CV), as well as glial fibrillary acidic protein (GFAP) immunostains, were obtained. For the present analyses, new microtome sections were cut from the available paraffin blocks and stained for additional markers according to routine diagnostic protocols, using a Ventana Benchmark ULTRA automated staining platform (Roche). The following primary antibodies were used: rabbit anti‐human GFAP (GA524, DAKO A/S, RRID:AB_2811722, 1:10′000), mouse anti‐human cluster of differentiation (CD)68 PG‐M1 (M0814, DAKO A/S, RRID:AB_2314148, 1:50), mouse anti‐human neurofilament 70/200 kDa 2F11/NE14 (M0762, DAKO A/S, RRID:AB_2314899, 1:50; MAB5256, SIGMA Chemical Company, RRID:AB_95184, 1:200), mouse anti‐human microtubule‐associated protein (MAP)2 HM‐2 (M9942, RRID:AB_477256, 1:30′000), rabbit anti‐human ubiquitin (Z0458, DAKO A/S, RRID:AB_2315524, 1:100), guinea pig anti‐human protein (p)62 (GP62‐C, PROGEN Biotech GmbH, RRID:AB_2687531, 1:12000), mouse anti‐human phosphorylated tau AT8 (MN1020, Thermofisher Scientific, RRID:AB_223647, 1:100), mouse anti‐human phosphorylated transactive response DNA binding protein (TDP)‐43 31F3 (CAC‐TIP‐PTD‐P03, Cosmo Bio, RRID:AB_1961901, 1:500), mouse anti‐human phosphorylated α‐synuclein (α‐syn) 5G4 (847‐0102004001, AJ‐RoboScreen GmbH, RRID:AB_NA, 1:500), mouse anti‐human amyloid beta (Aβ) A4G8 (800710, Biolegend, RRID:AB_2565324, 1:1500). Primary antibodies were detected with 3,3′‐diaminobenzidine (DAB; Ventana Optiview DAB IHC Detection Kit, Roche) or alkaline phosphatase red (Ventana UltraView Universal Alkaline Phosphatase Red Detection Kit, Roche). Slides were reviewed and imaged with a BX43 Manual System Olympus microscope and the cellSens entry software (Olympus) or with a Hamamatsu C9600 digital slide scanner (Hamamatsu Photonics).

### 2.6 Semi‐Quantitative Assessment of Neuronal Loss

Neuronal loss was assessed semi‐quantitatively in percentage categories, covering the heterogeneity of neuronal loss between different areas on HE stains and, when available, additionally on LFB‐CV stains. To ensure reproducibility, assessment was performed independently by three neuropathologists.

### 2.7 Statistics

Statistics and plots were performed in Excel (Microsoft, Version 2501) and MATLAB (MathWorks, Version 2022b), respectively.

### 2.8 Figure Design

Figures were designed in Affinity Designer 2 (Version 2.3.1).

## 3 Results

### 3.1 Clinical Features of McLeod Patients

Apart from one case, the main clinical findings of our patient cohort had been reported previously (Table 1). Initial presentations were variable and included movement disorders in three patients, muscular weakness or psychiatric symptoms in two patients each and splenomegaly in one patient. The mean age at symptom onset was 32.6 years (range 11–51 years). During the disease course, all patients developed a hyperkinetic, choreiform movement disorder (mean onset age 39.5 years, range 11–64 years), which was the initial symptom in three cases. Six patients suffered from psychiatric symptoms (mean onset age 42.8 years, range 25–60 years). In addition, six patients declined cognitively during the disease course (mean onset age 48.0 years, range 40–60 years). Epileptic seizures were not reported in any of the patients. Death occurred at a mean age of 57.5 years (range 50–69 years), after a mean disease duration of 24.9 years (range 14–46 years). Five patients died of cardiac complications, likely attributable to MLS, with intractable congestive heart failure in one patient and malignant arrhythmia and sudden cardiac death in four others. Two patients died of pneumonia, known as a complication of MLS [8]. For one patient, no information about the cause of death was available.

**TABLE 1:** Clinical features of McLeod patients.

Patient	Gender	Prior case report	Age at onset (years)	Initial clinical presentation	Cause of death (age)	Chorea onset (age)	Other movement disorders	Psychiatric symptoms onset (age)	Cognitive alterations onset (age)	Muscular involvement	Areflexia	Cardiopathy
1	M	Case 8 in Hewer et al. [7]	51	Weakness	Heart failure (69)	Yes (64)	No	No	No	UL none LL slight	UL LL	Dyspnoea Atrial fibrillation
2	M	Case IV‐13 in Jung et al. [5]	25	Personality disorder	SCD (55)	Yes (30)	Facial dyskinesia Dystonia	Personality disorder (25)	Yes (45)	UL slight LL moderate	UL LL	Cardiomyopathy
3	M	Case IV‐5 in Jung et al. [5]	39	Schizophrenia	SCD (55)	Yes (50)	Dysarthria	Schizophrenia (39)	Yes (48)	UL none LL slight	UL LL	No
4	M	Case IV‐6 in Jung et al. [5]	20	Chorea	Pneumonia (51)	Yes (20)	Dysarthria Feeding Dystonia	No	Yes (40)	UL moderate LL moderate	UL LL	No
5	M	—	30	Myoclonic jerks	SCD (58)	Yes (40)	Myoclonic jerks Dysarthria	OCD Bipolar disorder Anxiety Personality disorder (50)	Yes (55)	UL none LL slight	UL LL	SCD
6	M	Zeman et al. [19]	11	Restlessness Chorea Impulsivity/dysexecutive disorder	Pneumonia (57)	Yes (11)	Dysarthria	Compulsive collecting (40)	Yes (40)	No	UL LL	Cardiomyopathy
7	M	Case II‐4 in Symmans et al. [17]	34	Splenomegaly	SCD (50)	Yes (41)	Dysarthria	Exhibitionism (NA)	NA	UL slight LL slight	UL LL	Cardiomyopathy
8	M	Singleton et al. [18]	51	Leg weakness	Unknown (65)	Yes (60)	Motor and vocal tics	Personality disorder (60)	Yes (60)	UL and LL: severe with distal predominance	LL	Atrial fibrillation secondary to hyperthyroidism

Abbreviations: LL, lower limbs; NA, not available; OCD, obsessive–compulsive disorder; SCD, sudden cardiac death; UL, upper limbs.

### 3.2 Laboratory Findings of McLeod Patients

Laboratory findings are summarised in Table 2. Immunohaematological studies showed absent Kx RBC antigen and weak Kell antigens in all patients. Molecular genetic analyses of the *XK* gene revealed a nonsense pathogenic variant in one family, two cases with minor deletions of a single and of 13 base pairs (bp), respectively, a 7453 bp deletion of exon 2 in one case and major deletions of the entire *XK* gene in two other cases. All pathogenic variants predicted either an absent or a truncated XK protein devoid of the Kell protein binding site. One patient did not display erythrocyte acanthocytosis. In this case (Patient 5), repeated blood smears were not conducted, as the diagnosis had been confirmed by immunohaematological and molecular genetic studies. Serum CK levels were elevated in all patients (range 362–4000 U/L). One patient had an episode of severe rhabdomyolysis, possibly related to neuroleptic medication, with a rise of CK levels up to 160,820 U/L and subsequent renal failure [20].

**TABLE 2:** Laboratory, imaging and electrophysiology findings of McLeod patients.

Patient	Kell blood group phenotype	*XK* variant	RBC acanthocytes (%)	CK min–max (U/L)	ECG	Echo‐cardiography	EEG	EMG	ENG	Cerebral MRI/CT	Cerebral SPECT/PET
1	Kx 0, K1 0, K2 (+), K3 0, K4 (+), K5 (+), K6 0, K7 (+), K11 +, K12 +, K13 (+), K14 (+), K 18 (+), K17 0	1020‐1033del	8–85	1000	Atrial fibrillation LAFB	EF reduced enlargement LA and RV	NA	Neurogenic SA	Sensory‐motor axonal	Mild caudate nucleus and general atrophy	NA
2	Kx 0, K1 0, K2 (+), K3 0, K4 (+), K5 (+), K7 (+)	977C>T	20	3000	Normal SR	Normal	NA	NA	NA	Caudate nucleus and putamen atrophy	PET: decreased striatal FDG‐uptake
3	Kx 0, K1 0, K2 (+), K3 0, K4 (+), K5 (+), K7 (+)	977C>T	18	1500–160,820	Normal SR	Eccentric LVH EF 43%	NA	NA	NA	Mild caudate nucleus atrophy	PET: decreased striatal FDG‐uptake
4	Kx 0, K1 0, K2 (+), K3 0, K4 (+), K5 (+), K7 (+)	977C>T	20	362–5000	Normal SR	Mitral valve prolapse	NA	NA	NA	Caudate nucleus and putamen atrophy	PET: decreased striatal FDG‐uptake
5	Kx 0, K1 0, K2 0, K3 0, K4 0	Major deletion of the entire *XK* gene	0	400–4000	Normal SR	NA	Normal	Neurogenic SA	Motor axonal‐demyelinating	Increased proton density signal in basal ganglia	NA
6	Kx 0, K1 0, K2 (+), K3 0, K4 (+)	172delG	Yes (not specified)	1350	NA	NA	NA	NA	NA	Subtle, but definite caudate nucleus atrophy	SPECT: reduced Tc99m^x^ exametazime uptake caudate nucleus
7	Kx 0, K1 (+), K4 (+), K5 (+), K7 (+), K9 0, K13 (+)	Major deletion of the entire *XK* gene	30	788–3240	SR ectopic activity	LVH Enlargement LA and LV	NA	NA	NA	Enlargement of ventricles	NA
8	Kx 0, K1 0, K2 (+), K3 0, K4 (+), K5 (+), K6 (+)	7453 bp deletion, including exon 2	Yes (not specified)	431–2210	Atrial fibrillation	NA	Slowing due to sleepiness otherwise normal	Neurogenic SA	Sensory‐motor axonal	NA	NA

Abbreviations: bp, base pairs; C, cytosine; CK, creatine kinase; CT, computed tomography; del, deletion; ECG, electrocardiogram; EEG, electroencephalography; EF, ejection fraction; EMG, electromyography; ENG, electroneurography; FDG, fluorodeoxyglucose; G, guanine; LA, left atrium; LAFB, left anterior fascicular block; LV, left ventricle; LVH, left ventricular hypertrophy; MRI, magnetic resonance imaging; NA, not available; PET, positron emission tomography; RBC, red blood cell; RV, right ventricle; SA, pathological spontaneous activity; SPECT, single photon emission computed tomography; SR, sinus rhythm; T, thymine; Tc99m, technetium‐99‐molar.

### 3.3 Brain Imaging and Electrophysiology Findings of McLeod Patients

Neuroradiological examination of the brain was available for seven patients (Table 2). MRI (five patients) showed atrophy of the caudate nucleus of varying degrees (Table 2, Figure S1A). In addition, one patient showed mild global brain atrophy. PET of three patients revealed decreased striatal FDG uptake [5]. SPECT of another patient showed decreased perfusion of the caudate nucleus (Table 2, Figure S1B). EEGs (two patients) were without significant pathological findings. EMG and ENG (three patients) indicated axonal neuropathy, with signs of secondary demyelination in one case. ECG (seven patients) showed atrial fibrillation in two and marked ectopic activity in another patient. Echocardiography (five patients) revealed variable signs of cardiomyopathy in three cases and mitral valve prolapse in another.

### 3.4 Macroscopy of Different Brain Regions From McLeod Patients

**FIGURE 1:**
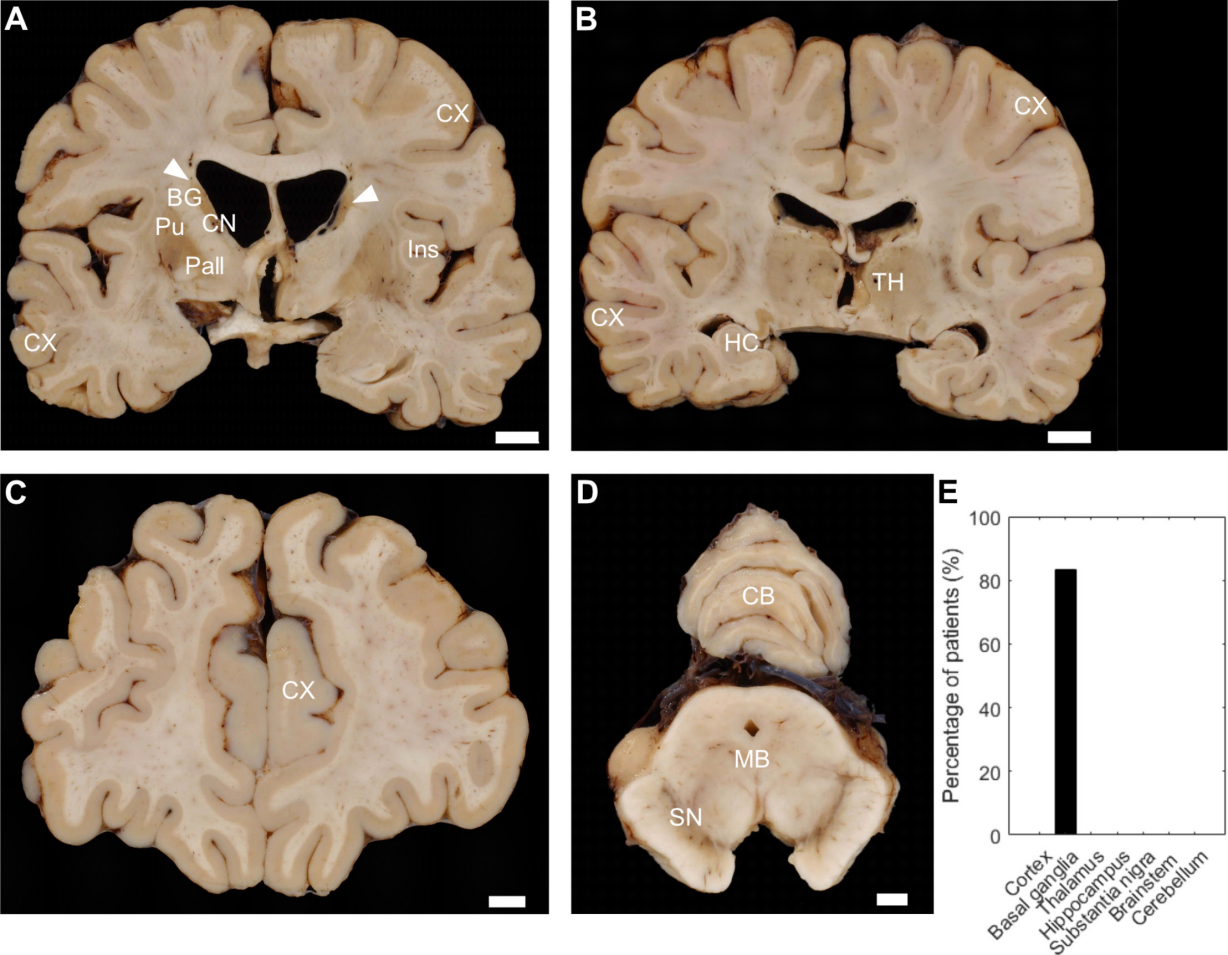
Macroscopy of different brain regions in McLeod patients. Coronal 7 mm thick brain slices, presenting (A) basal ganglia (BG) with severe atrophy of the basal ganglia, most prominent in the caudate nucleus (CN), with a beginning concave shape (arrowheads), and less evident in the putamen (Pu) and pallidum (Pall). The insula (Ins), frontotemporal cortex (CX), (B) thalamus (TH), hippocampus (HC), parietotemporal cortex (CX), (C) frontal cortex (CX), (D) midbrain (MB) with substantia nigra (SN) and cerebellum (CB) are inconspicuous. Scale bars: 1 cm. (E) Percentage of atrophy in distinct brain regions of McLeod patients.

Macroscopic findings documented by photography and/or written autopsy reports were available in six patients (Table S1). Autopsy findings of two patients (Patients 2 and 7) were previously published in abstract form and as two book chapters [12, 13, 16]. In the current case series, atrophy of the basal ganglia, particularly of the caudate nucleus, was evident in 83.3% (five of six) patients, ranging from mild to severe (Figure 1A,E, Table S1). Mild atrophy was characterised by slight enlargement of the lateral ventricles and subtle shrinkage of the caudate nucleus, which retained its convex shape. The putamen appeared largely unremarkable, and the pallidum was not visibly affected. Moderate atrophy correlated with further ventricular enlargement and more pronounced caudate volume loss, with transformation of the convex shape to a straight outline. Atrophy of the putamen was also evident, while the pallidum was visually inconspicuous. Severe atrophy was marked by extensive ventricular dilation and considerable reduction of the caudate nucleus, now exhibiting a concave shape. Besides more pronounced atrophy of the putamen, the pallidum also showed mild involvement. Very severe atrophy was defined by a markedly concave shape of the caudate nucleus, to the extent that the grey matter was scarcely visible. At this stage, atrophy of both the putamen and pallidum was clearly evident.

Besides mild brain oedema in a single patient, the remaining brain regions, including the substantia nigra, appeared unremarkable on gross examination (Figure 1B–E, Table S1).

In summary, atrophy of the basal ganglia, especially the caudate nucleus, was the most prominent macroscopic finding in our MLS patient cohort.

### 3.5 Histological Brain Examination of McLeod Patients

**FIGURE 2:**
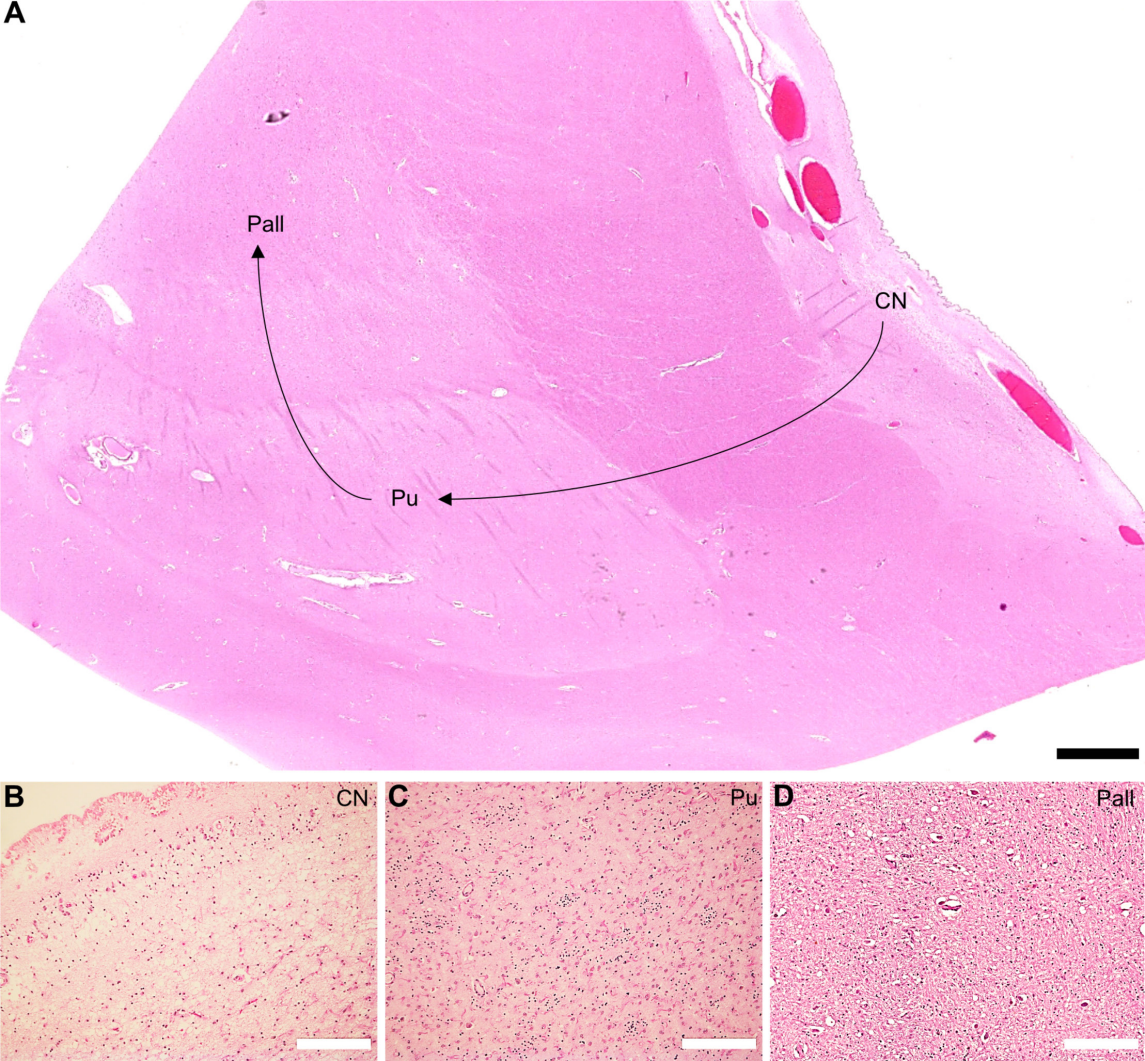
Histological findings in McLeod patients. (A) Overview haematoxylin and eosin (HE)‐stained slide of the basal ganglia, showing a gradient of neuronal loss and gliosis (arrows) from the caudate nucleus (CN) over the putamen (Pu) to the pallidum (Pall). (B–D) The gradient of neuronal loss in the caudate nucleus (B), putamen (C) and pallidum (D). Scale bars: 2 mm (A); 200 μm (B–D).

In all patients, we observed basal ganglia pathology, reflected by a gradient of neuronal loss accompanied by reactive gliosis and vacuolation of the neuropil. These findings were most prominent in the caudate nucleus, less prominent in the putamen and to an even lesser extent in the pallidum (Figure 2A–D). The degree of microscopic severity ranged from mild (< 25% neuronal loss; range in our cohort 10%–20%) to moderate (25%–50% neuronal loss; range in our cohort 30%–50%), severe (50%–80% neuronal loss; range in our cohort 60%–80%) and very severe (> 80% neuronal loss; range in our cohort 80%–90%). The pallidum was less involved and was even spared in some patients, who displayed only mild basal ganglia pathology (Figure S2, Table S2). In some cases, basal ganglia pathology was strikingly focal. In general, the severity of histological findings correlated with the degree of macroscopic atrophy (Tables S1 and S2).

**FIGURE 3:**
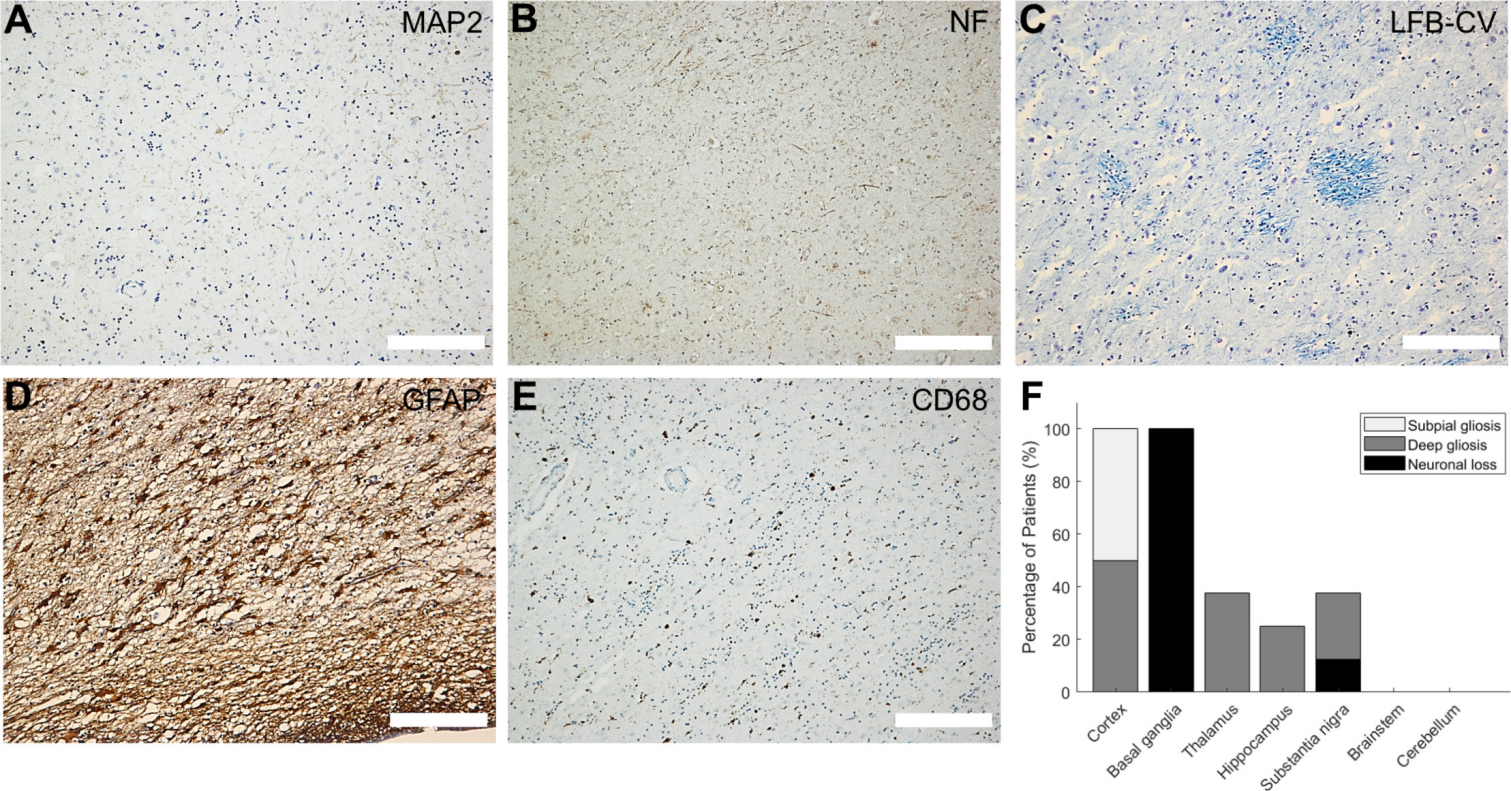
Special histological examination of the basal ganglia from McLeod patients. (A) MAP 2 immunohistochemistry, demonstrating neuronal loss. (B) Neurofilament (NF) immunohistochemistry to detect axonal loss. (C) Luxol fast blue‐cresyl violet (LFB‐CV) stain, displaying neuronal loss and preserved myelin. (D) GFAP immunohistochemistry, highlighting reactive astrocytosis. (E) CD68 immunohistochemistry, showing moderate microgliosis. Scale bars: 200 μm. (F) Percentage of neuronal loss and gliosis, respectively, in distinct brain regions of McLeod patients.

MAP 2 (Figure 3A) and neurofilament (Figure 3B) immunohistochemistry confirmed neuronal and axonal loss, respectively. Demyelination was not detected (Figure 3B,C). Gliosis was mainly characterised by the presence of reactive astrocytes and by less conspicuous microglial activation, as confirmed by immunohistochemistry for GFAP (Figure 3D) and CD68 (Figure 3E), respectively.

In addition, marked microangiopathic changes were observed on HE staining in the basal ganglia of two patients, as well as red, shrunken neurons as a sign of hypoxic damage in one who displayed global hypoxic neuronal damage in the brain (Table S2). However, because the topographic pattern of neuronal loss and gliosis in these patients cannot be explained by the aforementioned pathologies alone, these findings are likely unrelated to MLS.

Other brain regions were substantially less affected by neuronal loss and gliosis (Figures 3F and S3, Table S2). Single to numerous red, shrunken neurons on HE staining, as a sign of hypoxic neuronal damage of variable degree, were found in the cerebral cortex and the hippocampus of five patients, in the thalamus of one patient and the inferior olivary nucleus of another. Because these regions appeared largely unaffected in patients exhibiting pronounced basal ganglia pathology (Figure S3A–C, Table S2), we interpret these findings as unrelated to MLS. All cases showed subpial Chaslin's gliosis in the cortex that extended to superficial cortical layers in three and to deeper layers in one. In the substantia nigra, neuronal loss with gliosis was detected in one patient and isolated gliosis in two patients (Figure 3F, Table S2). However, there was no correlation between basal ganglia and substantia nigra pathology on visual inspection (Figure S3D, Table S2). We did not observe cerebellar pathology in any of the cases (Figure S3E, Table S2). Further brain regions were not systematically assessed.

Detailed histological examination at high magnification disclosed the presence of intravascular acanthocytes in the brains of three patients (Figure S3F, Table S2). As haemolysis impeded detailed RBC assessment, it is likely that acanthocytes in brain vessels are even more common in MLS.

In summary, we found histological evidence of basal ganglia pathology with neuronal loss and accompanying gliosis in all MLS cases of our series. Severity of involvement, however, varied considerably across the cases. The degree of macroscopic pathology correlated with the histological findings, with a decreasing gradient of severity from the caudate nucleus to the putamen and pallidum. Pathological findings in brain regions beyond the basal ganglia were non‐specific and inconsistent, and thus are likely unrelated to MLS.

### 3.6 Detection of Intraneuronal Vacuoles in the Basal Ganglia of McLeod Patients

**FIGURE 4:**
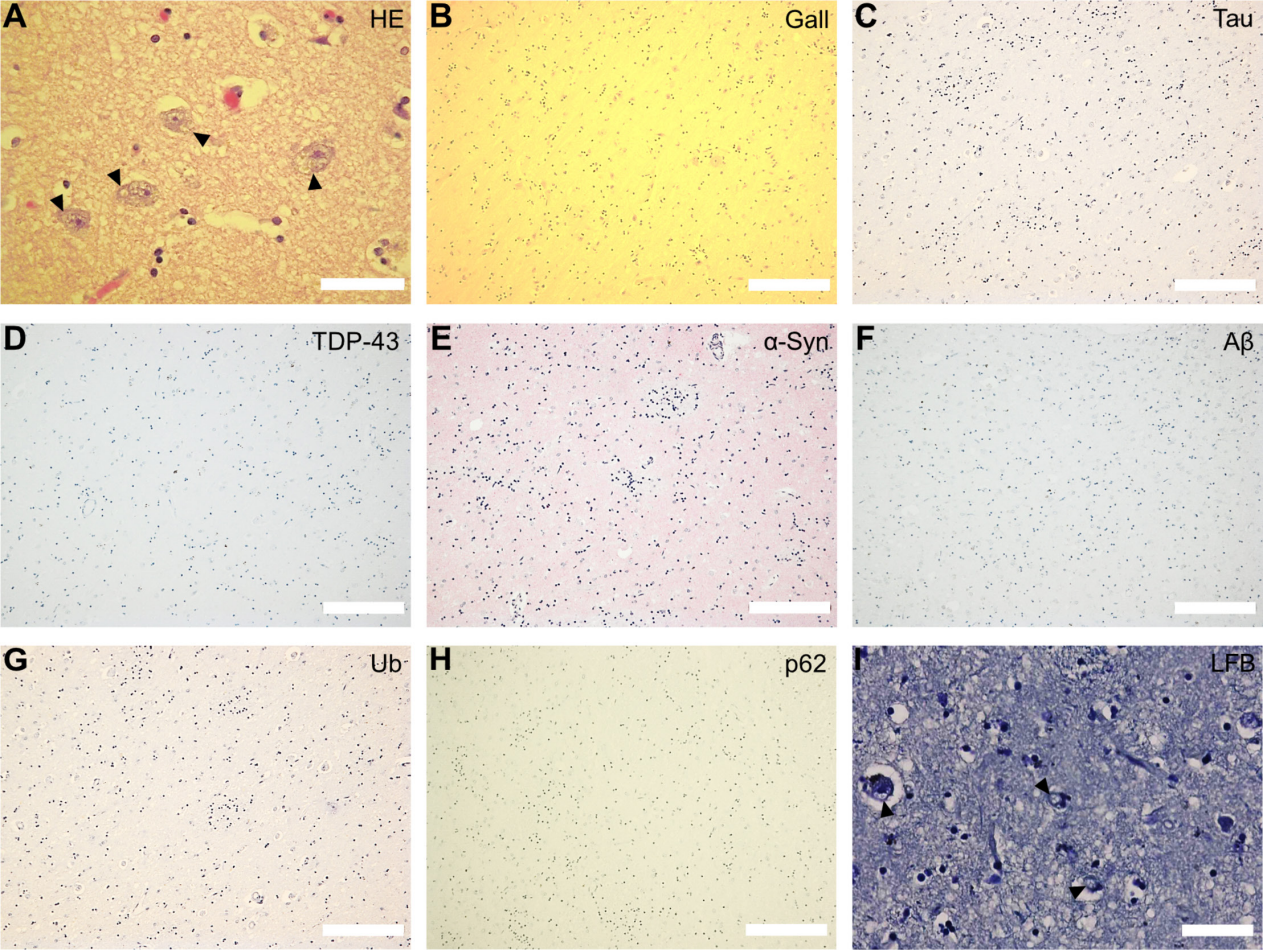
Intraneuronal cytoplasmic vacuoles in the basal ganglia of McLeod patients. (A) HE stain, revealing intraneuronal vacuoles (arrowheads). (B) Negative Gallyas silver (Gall) stain. (C–H) Negative immunohistochemistry for (C) hyperphosphorylated tau, (D) phosphorylated TDP‐43, (E) phosphorylated α‐synuclein (α‐syn), (F) amyloid beta (Aβ), (G) ubiquitin (Ub) and (H) p62. (I) LFB‐CV stain, not showing any lipid deposits within the vacuoles (arrowheads). Scale bars: 200 μm (B–H); 50 μm (A, I).

In addition to neuronal loss and gliosis‐associated vacuolation of the neuropil, we discovered intraneuronal cytoplasmic vacuoles in the basal ganglia in half of the cases (Figure 4A, Table S2). This finding was variable and ranged from focal vacuolation to moderate or even severe multivacuolation. Intraneuronal vacuoles were mainly detected in the caudate nucleus and putamen but were not observed in cases with only mild basal ganglia pathology. Protein aggregates were not detected in the basal ganglia of any of the MLS cases with Gall impregnation and immunohistochemistry for hyperphosphorylated tau, phosphorylated TDP‐43, phosphorylated alpha‐synuclein (α‐syn), Aβ, ubiquitin and p62 (Figure 4B–H). Isolated neurofibrillary tangles (NFTs) in the entorhinal cortex of the hippocampal region, consistent with a primary age‐related tauopathy (PART), Braak and Braak tau stage 1 [21], were observed in a single patient who had lived to the age of 58 years (Table S2). The discovery of XK's function as a lipid scramblase [10, 22] further raised the question about the presence of signs of lipid storage disease. However, no lipid deposits within the intraneuronal vacuoles were detected in the LFB stain (Figure 4I).

In summary, histological scrutiny of the basal ganglia revealed intraneuronal vacuoles in half of the cases. However, protein aggregates characteristic of neurodegenerative disorders or lipid deposits as a sign of lipid storage disease were not observed.

## 4 Discussion

In this study, we examined post‐mortem brain tissue from eight patients with MLS, constituting the largest neuropathological cohort of this extremely rare disease to date. Neuronal loss and concomitant reactive gliosis, more pronounced in the caudate nucleus and to a lesser extent in the putamen and pallidum, were consistent features. The degree of macroscopic atrophy correlated with the severity of histological alterations. Neuroimaging findings correlated with the neuropathology of MLS patients, also demonstrating a decreasing gradient of severity from the caudate nucleus to the putamen. Metabolic imaging indicated alterations in the striatum, such as reduced perfusion and decreased tracer uptake.

Our results confirm previous observations of single cases or small case series consisting of a maximum of three patients [11, 12, 13, 14, 23, 24, 25]. While the previous small case numbers did not allow for meaningful conclusions about a characteristic neuropathological pattern in MLS, the present study demonstrates for the first time a consistent pattern of brain involvement. This pattern appears centripetal, originating in the caudate, then spreading to affect deeper areas of the brain.

The pattern of brain involvement in MLS explains the distinctive clinical manifestations, with chorea as the cardinal symptom, as seen in other diseases marked by neuronal loss and gliosis in the basal ganglia in particular, striatal involvement in VPS13A disease and HD [24, 26]. Notably, VPS13A disease and MLS can be regarded as nearly identical phenocopies, with a slower disease progression in the latter [27]. The substantial clinical overlap between MLS and VPS13A disease is likely related to shared molecular pathways, which have been the focus of previous publications [27]. As a distinguishing feature, pathological changes in VPS13A disease are more pronounced in the thalamus. In one‐third of VPS13A patients developing parkinsonian features, additional nigral neuronal loss and gliosis of variable degree have been observed [23, 24, 26, 28, 29]. In contrast, neuropathological changes in the substantia nigra accompanied by parkinsonian features have been reported in only a minority of MLS patients [4, 5, 29]. In our cohort, the substantia nigra was variably affected in three out of eight cases (37.5%), but there was no correlation with the degree of basal ganglia pathology. Therefore, classification of nigral involvement in a temporal lesional pattern is difficult.

Based on our findings, the extension of basal ganglia pathology across the caudate nucleus to the putamen and finally to the pallidum appears across all MLS cases. As the pathological alterations are most pronounced in the caudate nucleus, this structure is probably affected at an early stage before progression to the putamen and pallidum. Therefore, analogous to the grading system for HD, which manifests a caudorostral, dorsoventral and mediolateral basal ganglia pathology [30, 31], we propose a similar neuropathological grading system for MLS. We have stratified the macroscopic and microscopic findings, i.e., atrophy and neuronal loss with astrogliosis, using a scoring system from zero to eight. Scoring is dependent on the degree of macroscopic and microscopic severity, with zero corresponding to normal, one to mild, two to moderate, three to severe and four to very severe basal ganglia pathology (Table 3).

**TABLE 3:** Proposed McLeod neuropathological grading system.

Basal ganglia macroscopy *(score)*	Basal ganglia microscopy *(score)*	Total score	Grade
Normal *(0)* to mild *(1)* atrophy (CN)	Mild neuronal loss and gliosis (CN > Pu) *(1)*	*1–2/8*	I
Mild *(1)* to moderate *(2)* atrophy (CN > Pu)	Moderate neuronal loss and gliosis (CN > Pu) *(2)*	*3–4/8*	II
Moderate *(2)* to severe *(3)* atrophy (CN > Pu)	Severe neuronal loss and gliosis (CN > Pu > Pall) *(3)*	*5–6/8*	III
Severe *(3)* to very severe *(4)* atrophy (CN > Pu > Pall)	Very severe neuronal loss and gliosis (CN > Pu > Pall) *(4)*	*7–8/8*	IV

*Note:* Macroscopic and microscopic scoring in italic brackets, total score in italic as number x from 8.

Abbreviations: CN, caudate nucleus; Pall, pallidum; Pu, putamen.

The proposed new grading system applied to our McLeod patient cohort is depicted in Table S3. Because our study was retrospective and detailed macroscopic findings were not available for all patients, neuroimaging findings were used as a surrogate for scoring purposes. Still, the limitation of macroscopic assessment was that the autopsy and neuroimaging reports were not standardised and often not precise.

The overall neuropathological severity grade is based on the total scores of both macroscopic and microscopic assessments, with total scores 1–2 corresponding to Grade I, 3–4 to Grade II, 5–6 to Grade III and 7–8 to Grade IV. In summary, Grade I represents macroscopically normal brain or only subtle caudate nucleus atrophy and histologically mild neuronal loss and gliosis in the caudate nucleus (as in Patients 1 and putatively 8). This stage, with only subtle macroscopic findings, may not be apparent on autopsy (as in Patient 1). Grade II describes mild macroscopic atrophy, more prominent in the caudate nucleus than the putamen, with moderate neuronal loss and gliosis of the caudate nucleus and, to a lesser extent, of the putamen on microscopic examination (as in Patients 3, 4 and 6). In Grade III, atrophy of the caudate nucleus is macroscopically moderate and more pronounced than in the putamen. Neuronal loss with gliosis is histologically severe in a gradient from the caudate nucleus over the putamen to the pallidum, the latter now also becoming involved (as in Patients 5 and 7). Finally, Grade IV is defined by severe macroscopic and very severe microscopic basal ganglia pathology (as in Patient 2). Grade IV+ will enable the addition of eventual pathological observations beyond the basal ganglia.

In our MLS patient cohort, we observed subpial Chaslin's cortical gliosis in all cases, with some showing extension to deeper cortical layers, but without any obvious correlation between the extent of subpial gliosis and the degree of basal ganglia pathology. With the addition of more MLS cases over time, this observation might become more meaningful and important for the neuropathological development of MLS.

Despite the clinical and neuropathological similarities between HD and MLS, the presence of specific inclusions in HD allows a clear neuropathological distinction between the two entities. While the neuropathology of HD can be explained by the accumulation of the neurotoxic mutant huntingtin protein [32], the exact molecular basis of MLS pathology in the brain has remained obscure. The current understanding links pathogenic variants in the *XK* gene with concomitant absence or dysfunction of the related XK protein to impaired lipid transportation in erythrocyte membranes [10, 33]. However, *XK* gene expression in most other cell types in MLS, as well as the predilection for the basal ganglia as the primarily affected CNS structure, is unclear [34, 35, 36]. In our study, we found intraneuronal cytoplasmic vacuoles in the basal ganglia of half of MLS patients, especially those with moderate to severe neuronal loss and gliosis. Hence, these vacuoles are likely to precede neuronal death, leading to selective MLS basal ganglia pathology. However, no disease‐defining protein aggregates were found on our histological examinations. In addition, no lipid deposits as a sign of lipid storage disease were detected within the intraneuronal vacuoles. Therefore, the content and the involved molecular pathways are not yet clear. Based on in vitro and HD mouse model results, Chhetri et al. suggested that impaired endosomal XK recycling for cellular manganese import promotes striatal vulnerability in HD [37]. Whether this hypothesis also applies to the vacuoles found in MLS patients requires further investigation.

In our study, detailed correlation with the clinical presentation was difficult due to the heterogeneity of available clinical descriptions. However, patients who initially presented at a more advanced age with mild symptoms of muscle weakness without any other strongly disabling movement disorders such as dysarthria, dystonia or dyskinesia, and died at a later age, showed only mild basal ganglia pathology without evidence of intraneuronal vacuoles (Table S4). Except for the subpial Chaslin's cortical gliosis found in all MLS patients, there was no obvious morphological correlate with the presence of cognitive and/or psychiatric symptoms. Thus, cognitive and psychiatric symptoms may rather be caused by disrupted functional connectivity of corticobasal ganglia pathways than by a morphological cortical pathology [38].

In summary, our investigation of the largest MLS patient cohort to date allows for the first time the delineation of a consistent pattern of neuropathological findings across all MLS cases from likely nonspecific co‐pathologies as seen in single patients. Our results indicate that the basal ganglia are the primarily affected brain structure, with decreasing severity from the caudate nucleus to the putamen and pallidum. Based on this observation, we propose a grading system for the neuropathological assessment of MLS, which is crucial for standardised examination of this extremely rare disease. Further validation in additional patients from all over the world is warranted, which remains a challenge due to the exceptional rarity of the disease.

Moreover, we discovered intraneuronal vacuoles in the basal ganglia of MLS patients with progressed neuropathology, which are likely to precede neuronal death. The nature of the intraneuronal vacuoles, as well as the underlying molecular mechanisms for the predilection of basal ganglia involvement in this rare genetic disease, remain to be elucidated.

## Author Contributions

A.M.R. and K.R. contributed equally.

Supply of patient material: F.G., J.H., P.F., B.U., A.Z. and R.R.R. Patient database and material management: A.M.R., K.R. and H.H.J. Ethical permit writing and submission: A.M.R., K.R. and H.H.J. Clinical data assessment: K.R. and H.H.J. Neuropathological assessment: A.M.R., T.H. and E.J.R. Statistics: A.M.R. Preparation of figures and tables: A.M.R. and K.R. Manuscript writing: A.M.R. and K.R. with comments from T.H., F.G., a.d., P.F., A.Z., E.J.R. and H.H.J. Study supervision: H.H.J.

All authors made substantial contributions to the conception or design of the study, or to the acquisition, analysis or interpretation of data. All authors approved the manuscript.

## Ethics Statement

This study was approved by the local ethics committee in Zurich (KEK; BASEC‐Nr. 2022‐006) in accordance with the Declaration of Helsinki. Because consent to participate could not be obtained after death, the biological material for the study was surplus material in the framework of autopsy diagnostics; due to the different standards in the different countries from which the material was obtained and due to sample collection over a long period of 8 years with changing standards, the study was approved without the need for consent.

## Conflicts of Interest

The authors declare no conflicts of interest.

## Supporting information


**Figure S1:** Neuroradiology of McLeod patients. (A) MRI, showing atrophy of the basal ganglia, particularly of the caudate nucleus (arrowheads). (B) Tc99m exametazime SPECT, revealing reduced perfusion of the caudate nucleus (arrowheads).
**Figure S2:** Histological severity degree assessment of neuronal loss and gliosis in McLeod patients with haematoxylin and eosin (HE) stains. (A–C) Mild, (D–F) moderate, (G–I) severe and (J–L) very severe basal ganglia pathology, reflected by gradients of neuronal loss and gliosis from the caudate nucleus (CN) over the putamen (Pu) to the pallidum (Pall). Scale bars: 200 μm.
**Figure S3:** Histology of other brain regions and brain acanthocytes in McLeod patients. Haematoxylin and eosin (HE)‐stained sections from (A) frontal cortex (CX), (B) hippocampus (HC), (C) thalamus (TH), (D) substantia nigra (SN) of the midbrain and (E) cerebellum (CB), not indicating any specific pathology. (F) HE‐stained section, showing acanthocytes (arrowheads) in cerebral vessels. Scale bars: 200 μm (A–E); 50 μm (F).
**Table S1:** Macroscopy of different brain regions from McLeod patients.
**Table S2:** Histology of distinct brain regions from McLeod patients.
**Table S3:** The proposed grading system applied to the McLeod patient cohort.
**Table S4:** The proposed grading system in the McLeod patient cohort with clinical correlations.

## Data Availability

The raw data supporting the conclusions of this article are provided within the article and its Supporting Information. Due to data privacy regulations, patient reports and the university hospital database cannot be publicly shared. Interested parties may request access by contacting the corresponding author. Upon such requests, a data‐sharing agreement can be established between our host institution (University Hospital Zurich) and the inquiring party.
