# Supplementary material for: Neuropathological Characterisation of McLeod Syndrome With a Proposed New Grading System
Source: Neuropathol Appl Neurobiol. 2025 Sep 2;51(5):e70039. doi: 10.1111/nan.70039 (PMC12547491; doi:10.1111/nan.70039)
Supplement: Supplementary file 1 — Figure S1: Neuroradiology of McLeod patients. (A) MRI, showing atrophy of the basal ganglia, particularly of the caudate nucleus (arrowheads). (B) Tc99m exametazime SPECT, revealing reduced perfusion of the caudate nucleus (arrowheads). Figure S2: Histological severity degree assessment of neuronal loss and gliosis in McLeod patients with haematoxylin and eosin (HE) stains. (A–C) Mild, (D–F) moderate, (G–I) severe and (J–L) very severe basal ganglia pathology, reflected by gradients of neuronal loss and gliosis from the caudate nucleus (CN) over the putamen (Pu) to the pallidum (Pall). Scale bars: 200 μm. Figure S3: Histology of other brain regions and brain acanthocytes in McLeod patients. Haematoxylin and eosin (HE)‐stained sections from (A) frontal cortex (CX), (B) hippocampus (HC), (C) thalamus (TH), (D) substantia nigra (SN) of the midbrain and (E) cerebellum (CB), not indicating any specific pathology. (F) HE‐stained section, showing acanthocytes (arrowheads) in cerebral vessels. Scale bars: 200 μm (A–E); 50 μm (F). Table S1: Macroscopy of different brain regions from McLeod patients. Table S2: Histology of distinct brain regions from McLeod patients. Table S3: The proposed grading system applied to the McLeod patient cohort. Table S4: The proposed grading system in the McLeod patient cohort with clinical correlations. [file NAN-51-e70039-s001.docx]

**Supplementary Information**

**Neuropathological characterisation of McLeod Syndrome
with a proposed new grading system**

Anna Maria Reuss ^1)^*, Klavs Renerts^2)*^, Tibor Hortobágyi^1)^, Felix Geser^1)^, Johannes Haybaeck^1)^, Adrian Danek^3)^, Peter Fuhr^4)^, Bjarne Udd^5)^, Adam Zeman^6,7)^, Reichard R. Ross^8)^, Elisabeth J. Rushing^1)^, Hans H. Jung^2)^

*contributed equally

1) Institute of Neuropathology, University Hospital Zurich, University of Zurich, Switzerland

2) Department of Neurology, University Hospital Zurich, University of Zurich, Switzerland

3) Department of Neurology, Ludwig-Maximilian-University (LMU) University Hospital Munich, Germany

4) Depts. of Neurology and of Clinical Research, University Hospital Basel, Switzerland

5) Department of Neurology, Tampere University Hospital and Vasa Central Hospital, Finland

6) Department of Clinical Neurosciences, University of Edinburgh

7) University of Exeter Medical School, United Kingdom

8) Department of Laboratory Medicine and Pathology, Mayo Clinic, Rochester MN, USA

Correspondence to:

Prof. Dr. med. Hans H. Jung, MD

E-Mail: hans.jung@usz.ch

**Supplementary Figures**


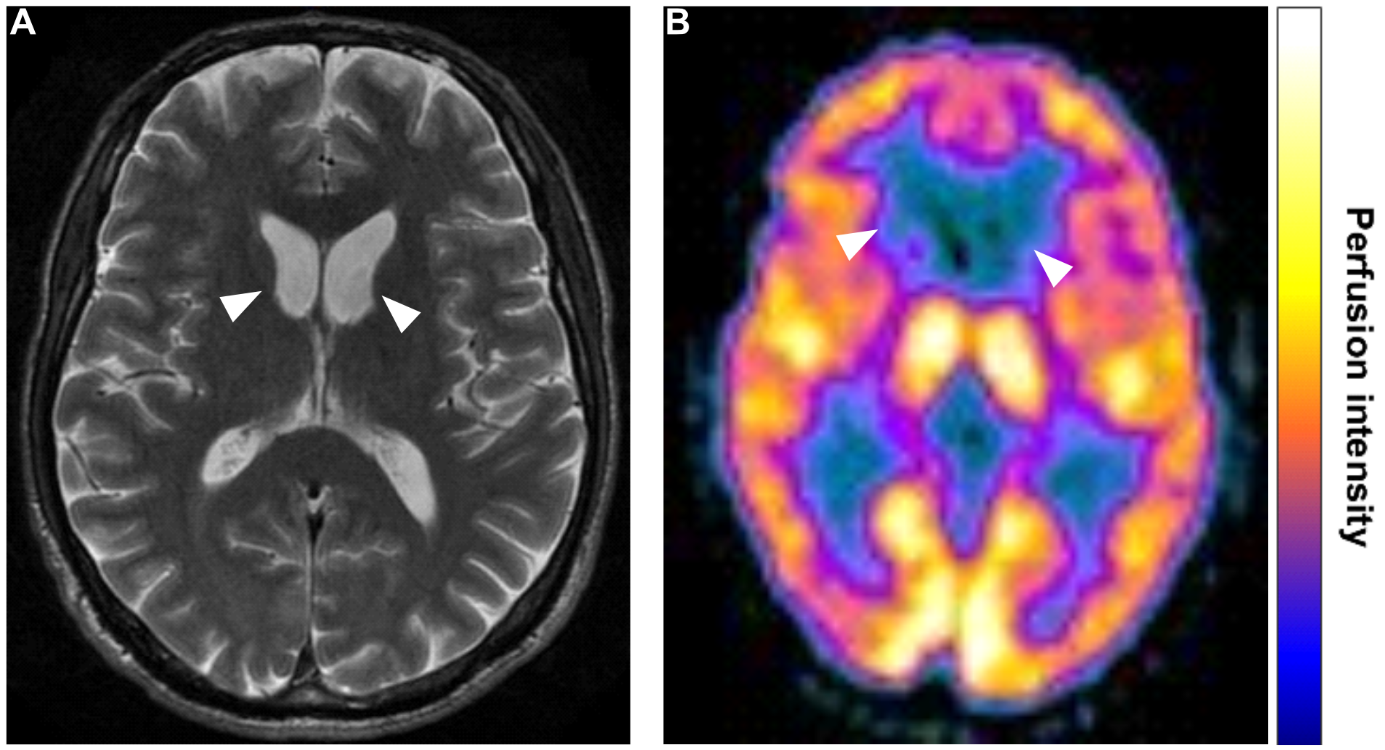


Figure S1 Neuroradiology of McLeod patients. (A) MRI, showing atrophy of the basal ganglia, particularly of the caudate nucleus (arrowheads). (B) Tc99m exametazime SPECT, revealing reduced perfusion of the caudate nucleus (arrowheads).


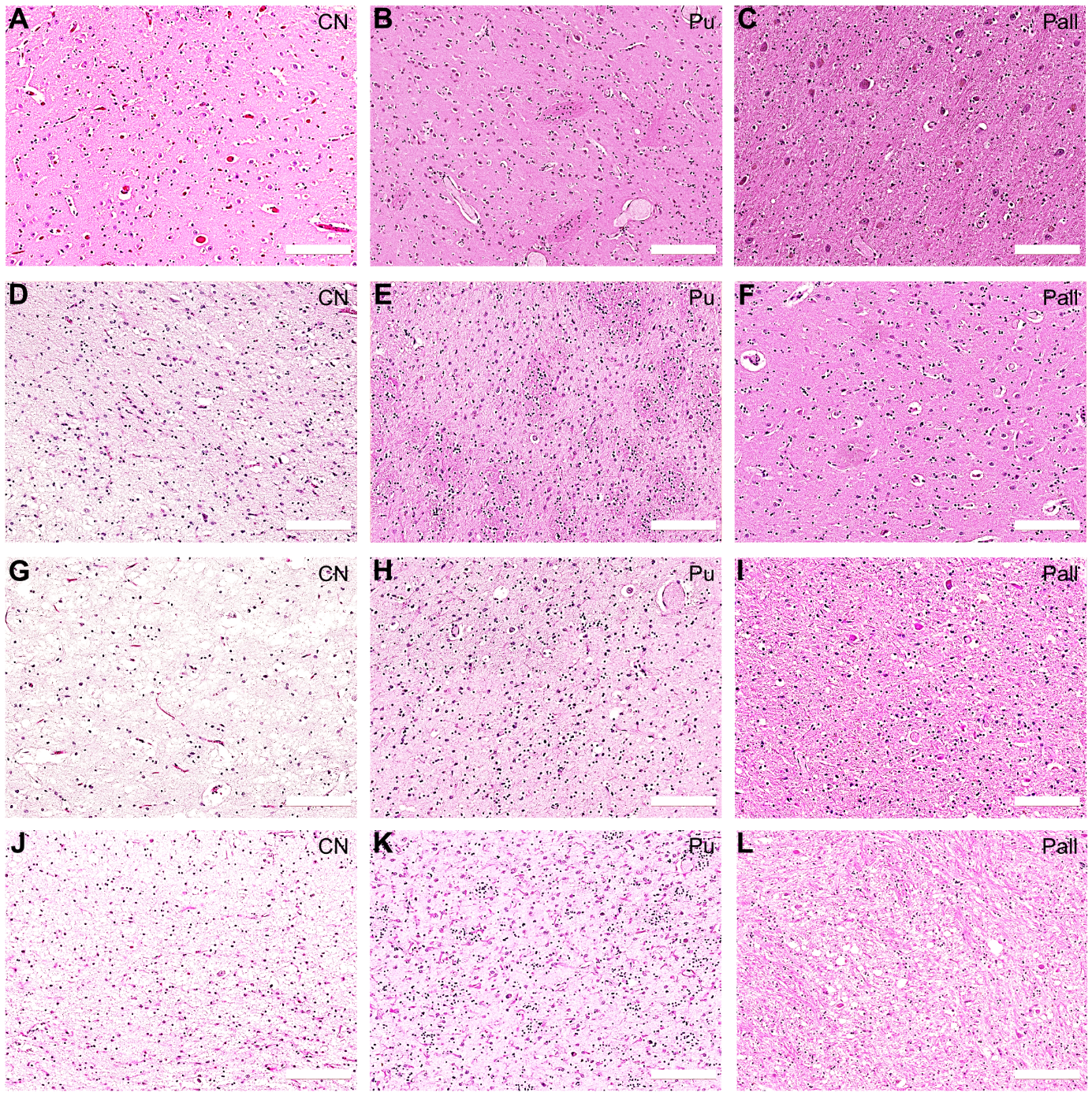


Figure S2 Histological severity degree assessment of neuronal loss and gliosis in McLeod patients with haematoxylin and eosin (HE) stains. (A-C) Mild, (D-F) moderate, (G-I) severe, and (J-L) very severe basal ganglia pathology, reflected by gradients of neuronal loss and gliosis from the caudate nucleus (CN) over the putamen (Pu) to the pallidum (Pall). Scale bars: 200 µm.


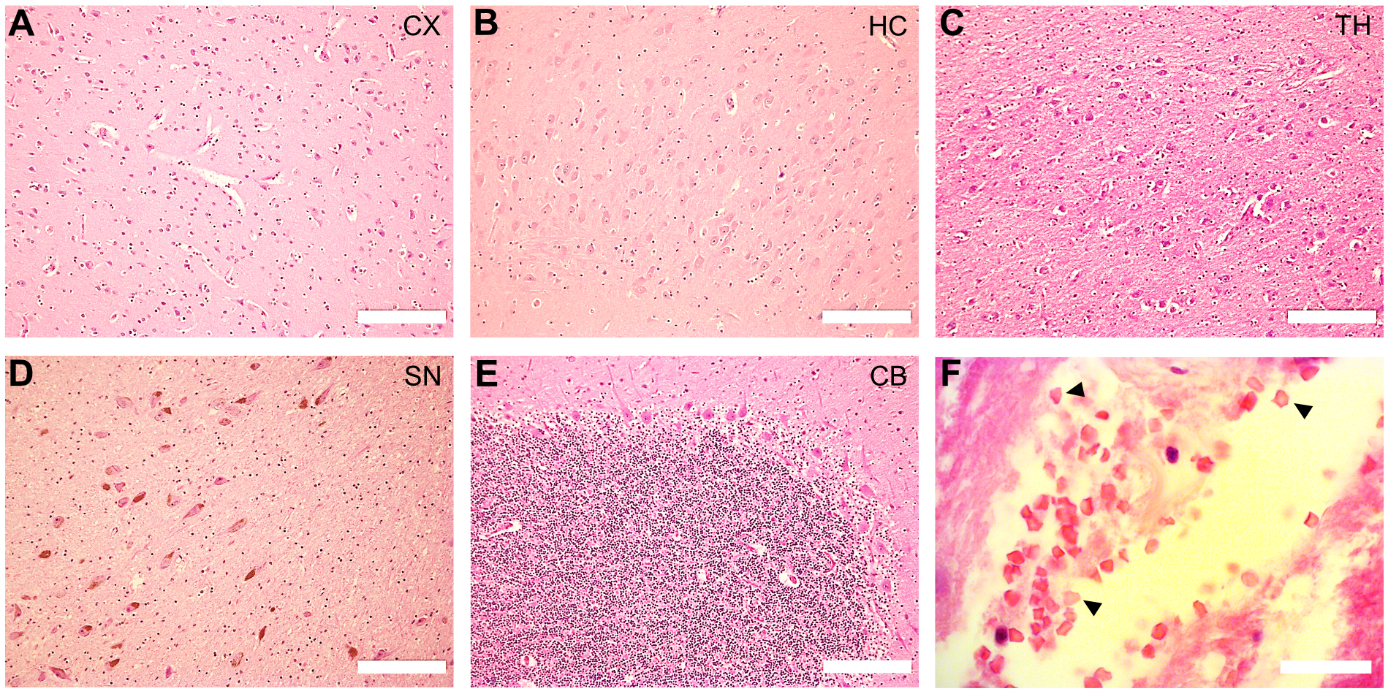


Figure S3 Histology of other brain regions and brain acanthocytes in McLeod patients. Haematoxylin and eosin (HE)-stained sections from (A) frontal cortex (CX), (B) hippocampus (HC), (C) thalamus (TH), (D) substantia nigra (SN) of the midbrain, and (E) cerebellum (CB), not indicating any specific pathology. (F) HE-stained section, showing acanthocytes (arrowheads) in cerebral vessels. Scale bars: 200 µm (A-E); 50 µm (F).

**Supplementary Tables**

**Table S1** Macroscopy of different brain regions from McLeod patients

| Patient | Cortex | Basal ganglia | Thalamus | Hippocampus | Substantia nigra | Brainstem | Cerebellum |
| --- | --- | --- | --- | --- | --- | --- | --- |
| 1 | Normal | Normal | Normal | Normal | Normal | Normal | Normal |
| 2 | Normal | Severe atrophy (CN > Pu > Pall) | Normal | Normal | Normal | Normal | Normal |
| 3 | Normal | Mild atrophy (CN > Pu) | Normal | Normal | Normal | Normal | Normal |
| 4 | Normal | Mild atrophy | Normal | Normal | Normal | Normal | Normal |
| 5 | Mild brain oedema | Moderate atrophy (CN and Pu) | Normal | Normal | Normal | Normal | Normal |
| 6 | NA | NA | NA | NA | NA | NA | NA |
| 7 | Normal | Moderate atrophy (CN) | Normal | Normal | Normal | Normal | Normal |
| 8 | NA | NA | NA | NA | NA | NA | NA |

Abbreviations: CN: caudate nucleus, Pu: putamen, Pall: pallidum, NA: not available

**Table S2** Histology of distinct brain regions from McLeod patients

| Patient | Cortex | Basal ganglia | Thalamus | Hippocampus | Substantia nigra | Brainstem | Cerebellum |
| --- | --- | --- | --- | --- | --- | --- | --- |
| 1 | Mild hypoxia and subpial gliosis | Mild neuronal loss and gliosis (CN > Pu), acanthocytes | Mild gliosis | Mild hypoxia and gliosis | Normal | Hypoxia in ION | Normal |
| 2 | Mild hypoxia and gliosis | Very severe neuronal loss and gliosis (CN > Pu > Pall), few vacuoles, acanthocytes | Normal | Mild hypoxia | Normal | Calcifications perivascular and in ION | Normal |
| 3 | Mild subpial gliosis | Moderate neuronal loss and gliosis (CN > Pu), multivacuoles | Normal | Mild hypoxia and gliosis | Mild gliosis | Normal | Normal |
| 4 | Mild hypoxia and gliosis of superficial layers | Moderate neuronal loss and gliosis (CN > Pu), few vacuoles, acanthocytes | Normal | Mild hypoxia | Normal | Normal | Normal |
| 5 | Mild hypoxia, oedema, gliosis of superficial layers | Severe neuronal loss and gliosis (CN > Pu > Pall), multivacuoles, marked microangiopathy | Mild gliosis | Single NFT  in EC | Normal | Normal | Normal |
| 6 | Hypoxia and gliosis of superficial layers | Moderate neuronal loss and gliosis (CN > Pu), hypoxia | Mild hypoxia and gliosis | Hypoxia | Gliosis | Normal | Normal |
| 7 | Subpial gliosis | Severe neuronal loss and gliosis (CN > Pu > Pall) | Normal | Normal | Severe neuronal loss and gliosis | Normal | Normal |
| 8 | Subpial gliosis | Mild neuronal loss and gliosis (CN > Pu), marked microangiopathy | Normal | Normal | Normal | Normal | Normal |

Abbreviations: CN: caudate nucleus, Pu: putamen, ION: inferior olivary nucleus, Pall: pallidum, NFT: neurofibrillary tangles, EC: entorhinal cortex

**Table S3** The proposed grading system applied to the McLeod patient cohort

| Patient | Cerebral MRI/CT *(score)* | Basal ganglia macroscopy *(score)* | Basal ganglia microscopy *(score)* | Total score | Grade *(total score)* |
| --- | --- | --- | --- | --- | --- |
| **1** | Mild CN and general atrophy *(1)* | Normal *(0)* | Mild neuronal loss and gliosis (CN > Pu), acanthocytes *(1)* | *2/8* | I *(1-2/8)* |
| 2 | CN and Pu atrophy | Severe atrophy (CN > Pu > Pall) *(3)* | Very severe neuronal loss and gliosis (CN > Pu > Pall), few vacuoles, acanthocytes *(4)* | *7/8* | IV *(7-8/8)* |
| 3 | Mild CN atrophy | Mild atrophy (CN > Pu) *(1)* | Moderate neuronal loss and gliosis (CN > Pu), multivacuoles *(2)* | *3/8* | II *(3-4/8)* |
| 4 | CN and Pu atrophy | Mild atrophy *(1)* | Moderate neuronal loss and gliosis (CN > Pu), few vacuoles, acanthocytes *(2)* | *3/8* | II *(3-4/8)* |
| 5 | Increased proton density signal in basal ganglia | Moderate atrophy (CN and Pu) *(2)* | Severe neuronal loss and gliosis (CN > Pu > Pall), multivacuoles *(3)* | *5/8* | III *(5-6/8)* |
| 6 | Subtle, but definite CN atrophy *(1)* | NA | Moderate neuronal loss and gliosis (CN > Pu) *(2)* | *3/8* | II *(3-4/8)* |
| 7 | Enlargement of ventricles | Moderate atrophy (CN) *(2)* | Severe neuronal loss and gliosis (CN > Pu > Pall) *(3)* | *5/8* | III *(5-6/8)* |
| 8 | NA | NA | Mild neuronal loss and gliosis (CN > Pu) *(1)* | *1/8* | I *(1-2/8)* |

Abbreviations: MRI: magnetic resonance imaging, CT: computed tomography, CN: caudate nucleus, Pu: putamen, Pall: pallidum, NA: not available;

Macroscopic and microscopic scoring in italic brackets, total scores in italic as number x from eight

**Table S4** The proposed grading system in the McLeod patient cohort with clinical correlations

| Patient | Grade *(total score)* | **Intraneuronal vacuoles** | **Age at**  onset (years) | **Age at death**  (years) | Years from onset to death | Initial clinical presentation | Other movement disorders | **Psychiatric symptoms onset (age)** |
| --- | --- | --- | --- | --- | --- | --- | --- | --- |
| **1** | I *(1-2/8)* | No | 51 | 69 | 18 | Weakness | No | No |
| 2 | IV *(7-8/8)* | Few | 25 | 55 | 30 | Personality disorder | Facial dyskinesia  Dystonia | Personality disorder  (25) |
| 3 | II *(3-4/8)* | Multivacuoles | 39 | 55 | 16 | Schizophrenia | Dysarthria | Schizophrenia  (39) |
| 4 | II *(3-4/8)* | Few | 20 | 51 | 31 | Chorea | Dysarthria  Feeding  Dystonia | No |
| 5 | III *(5-6/8)* | Multivacuoles | 30 | 58 | 28 | Myoclonic jerks | Myoclonic jerks  Dysarthria | OCD  Bipolar disorder  Anxiety  Personality disorder  (50) |
| 6 | II *(3-4/8)* | No | 11 | 57 | 46 | Restlessness  Chorea  Impulsivity/dysexecutive disorder | Dysarthria | Compulsive collecting (40) |
| 7 | III *(5-6/8)* | No | 34 | 50 | 16 | Splenomegaly | Dysarthria | Exhibitionism (NA) |
| 8 | I *(1-2/8)* | No | 51 | 65 | 14 | Leg weakness | Motor and vocal tics | Personality disorder  (60) |

Abbreviations: OCD: obsessive compulsive disorder, NA: not available; Total scores in italic as number x from eight
